# Family-based gene-environment interaction using sequence kernel association test (FGE-SKAT) for complex quantitative traits

**DOI:** 10.1038/s41598-021-86871-2

**Published:** 2021-04-01

**Authors:** Chao-Yu Guo, Reng-Hong Wang, Hsin-Chou Yang

**Affiliations:** 1 Division of Biostatistics, Department of Medicine, Institute of Public Health, School of Medicine, National Yang-Ming University, Taipei, Taiwan; 2 Institute of Public Health, School of Medicine, National Yang Ming Chiao Tung University, Hsinchu, Taiwan; 3 Institute of Statistical Science, Academia Sinica, Taipei, Taiwan

**Keywords:** Computational biology and bioinformatics, Computational models, Genome informatics, Hardware and infrastructure, Statistical methods

## Abstract

After the genome-wide association studies (GWAS) era, whole-genome sequencing is highly engaged in identifying the association of complex traits with rare variations. A score-based variance-component test has been proposed to identify common and rare genetic variants associated with complex traits while quickly adjusting for covariates. Such kernel score statistic allows for familial dependencies and adjusts for random confounding effects. However, the etiology of complex traits may involve the effects of genetic and environmental factors and the complex interactions between genes and the environment. Therefore, in this research, a novel method is proposed to detect gene and gene-environment interactions in a complex family-based association study with various correlated structures. We also developed an R function for the Fast Gene-Environment Sequence Kernel Association Test (FGE-SKAT), which is freely available as supplementary material for easy GWAS implementation to unveil such family-based joint effects. Simulation studies confirmed the validity of the new strategy and the superior statistical power. The FGE-SKAT was applied to the whole genome sequence data provided by Genetic Analysis Workshop 18 (GAW18) and discovered concordant and discordant regions compared to the methods without considering gene by environment interactions.

## Introduction

After the genome-wide association studies^1–6^, common genetic markers associated with complex diseases and quantitative traits have been successfully identified. However, so far, for most complex diseases and quantitative traits, all identified genetic markers can only explain a small proportion of genetic components of complex diseases and quantitative traits, suggesting that there are still missing heritability to be discovered by genetic markers.

Genome-wide association studies have focused on the genetic association of common variants with complex diseases. However, rare variants may also play a key role in influencing certain complex diseases and traits^7^ and explain additional disease risks or traits of heritability. A rare variation is usually defined as the minor allele frequency (MAF) < 0.5%.

With the advances in sequencing technology, new and useful whole-exome sequencing has been developed. As a result, robust and efficient statistical methods for the association between rare variants and complex diseases and traits are desired.

A conventional association test uses one genetic marker at one time to identify common variations that are associated with a disease or trait. Although one could repeatedly implement the simple technique to discover some rare variations, this approach suffers an insufficient statistical power after adjusting for multiple testing. As a result, the decision of the overall statistical testing is too conservative^8,9^. Therefore, the genomic region-based assessment considers multiple variants and traits, such as the collapsing method^10^ and the sequence kernel association test (SKAT)^11^, a flexible and efficient regression method for the associations between genomic regions and quantitative traits with consideration of covariates. The SKAT is based on a mixed effect model and overcomes the power issue in the collapsing method, especially when the genetic effects are positive in some variants and negative in the other ones. The kernel function interprets the gene segment, the random effect in the mixed effect model. If the subjects are correlated with family structures, the fast family-based SKAT (FFBSKAT) was developed to avoid invalid results^12,13^.

In genetic studies, common variations could partially explain most diseases. SNPs may affect traits, but the environmental factors may modify the effect of SNPs. Tests for gene-environment interactions using one SNP and one environmental factor have been proposed^14^. For better statistical power, such types of interactions using genomic regions are also discussed^15,16^. Recent studies also showed surprising findings with gene-environment interactions^17,18^.

This research aims to develop a novel and efficient statistical model for the genomic region-based assessment using multiple variants and traits to test gene-environment interactions under the complex familiar structures. Therefore, we extend the SKAT model under the family-based design to identify gene-environment interactions and the gene effect, named the Fast Gene-Environment Sequence Kernel Association Test (FGE-SKAT). Simulation studies with ten thousand repetitions confirm the validity of the new strategy. In addition to simulation studies, the new strategy is applied to the whole genome sequence data from the Genetic Analysis Workshop 18 (GAW18). Finally, a freely available R^19^ function with a detailed manual and an automatic pipeline for GWAS are ready for easy implementation of the new method FGE-SKAT, where six essential packages are also integrated, including "CompQuadForm", "kinship2", "SKAT", "survey", "rareGE", "ggplot2", and "quadprog".

## Materials and methods

Following the previous works^12,13,16,20^, the inheritance of a quantitative trait with gene-environment interactions in the sample of "n" genetically related subjects could be presented in a linear mixed effect model, which is defined as $y = X\alpha + h_{{1}} + h_{2} + b + \varepsilon$. The symbol $y$ denotes the $n \times 1$ vector of phenotypes and $X$ is the $n \times p$ matrix of covariates,$\alpha$ is the $p \times 1$ matrix of regression coefficients of the covariates. $h_{1}$,$h_{2}$, $b$ and $\varepsilon$ are $n \times 1$ vectors of random effects for gene, gene-environment interaction, family effect, and random error, respectively. $h_{1}$ is assumed to follow a normal distribution, $\mathrm{N}(0,{\tau }_{1}{K}_{1})$. ${\text{K}}_{{1}}$ is the $n \times n$ matrix with elements defined by the kernel function of individual phenotypes in the region to be analyzed. $\tau_{1}$ is the variance component representing the correlations resulting from the regional genotypes. ${\text{K}}_{{1}}$ is also called the weighted linear kernel function, defined as ${\text{K}}_{{1}} = \rho {\text{GWWG}}^{{\text{T}}}$, where $G$ denotes the $n \times m$ matrix of individual genotypes in the region to be analyzed, m is the number of SNPs, W is the $m \times m$ diagonal matrix of SNP weights. $h_{2}$ follows $\mathrm{N}(0,{\tau }_{2}{K}_{2})$, ${\text{K}}_{{2}}$ is the $n \times n$ kernel function representing gene-environment interactions and ${\text{K}}_{{2}} = {(1 - }\rho {\text{)EGWWG}}^{{\text{T}}} {\text{E}}$, E is the $n \times n$ diagonal matrix of the environment factor^16^. Vector $b$ is assumed to be distributed as normal $N(0,\sigma_{b}^{2} R)$, where R is the $n \times n$ relationship (twice kinship) matrix, $\sigma_{b}^{2}$ is the variance component that models within-family correlations, $\varepsilon$ is $N(0,\sigma_{e}^{2} I_{n} )$, and $I_{n}$ is the $n \times n$ identity matrix, $\sigma_{e}^{2}$ is the variance component of random errors.

Let ${\text{K}} = K_{{1}} + K_{{2}}$, then ${\text{K}} = K_{{1}} + K_{{2}} = \rho {\text{GWWG}}^{{\text{t}}} + {(1 - }\rho {\text{)EGWWG}}^{{\text{t}}} {\text{E}}$. When ${\text{H}}_{{0}} {:}\tau = {0}$, ${\text{V}} = \sigma_{b}^{2} R + \sigma_{e}^{2} I_{n}$, where $\sigma_{b}^{2}$, $\sigma_{e}^{2}$, and $\alpha$ could be obtained by the Maximum likelihood estimation^12^. Note that $\alpha = (X^{T} {\text{V}}^{{ - 1}} X)^{ - 1} X^{T} {\text{V}}^{{ - 1}} y$.

In the mixed effect model, $$\tau$$ is estimated by the restricted maximum likelihood (REML) estimation to limit the potential bias^12,13^. The likelihood function is $$l = - 0.5\ln |V| - 0.5\ln |X^{T} V^{ - 1} X| - 0.5(y - X\hat{\alpha })^{T} V^{ - 1} (y - X\hat{\alpha })$$. The score test under $${\text{H}}_{{0}} {:}\tau = {0}$$ is derived by the partial derivative of $$\tau$$, where $$\frac{\partial l}{{\partial \tau }} = - 0.5tr(V^{ - 1} - V^{ - 1} X(X^{T} V^{ - 1} X)^{ - 1} X^{T} V^{ - 1} )\frac{\partial V}{{\partial \tau }} + 0.5(y - X\overset{\lower0.5em\hbox{$\smash{\scriptscriptstyle\frown}$}}{\alpha } )^{T} V^{ - 1} {\text{K}}V^{ - 1} (y - X\overset{\lower0.5em\hbox{$\smash{\scriptscriptstyle\frown}$}}{\alpha } )$$. Detailed proofs are in Supplementary Materials. Following the Previous works^11–13,15,21–24^, test statistics of the FGE-SKAT are based on the second term of the likelihood function.

In the proposed model, the quantitative trait follows a multivariate normal distribution with the vector of means $X\alpha$ and the covariance matrix $\sigma_{{\text{b}}}^{{2}} {\text{R}} + \tau_{{1}} K_{{1}} + \tau_{{2}} K_{{2}} + \sigma_{e}^{2} I_{n}$. Under the null hypothesis (${\text{H}}_{{0}} {:}\tau_{1} = \tau_{2} = {0}$), the covariance matrix becomes ${\text{V}} = \sigma_{b}^{2} R + \sigma_{e}^{2} I_{n}$, $\alpha = (X^{T} {\text{V}}^{{ - 1}} X)^{ - 1} X^{T} {\text{V}}^{{ - 1}} y$. The score statistics is ${\text{Q}} = {{0.5\{ ({\text{y}} - {\text{X}}}}\alpha {)}^{{\text{T}}} {\text{V}}^{{ - 1}} {\text{KV}}^{{ - 1}} {{({\text{y}} - {\text{X}}}}\alpha {{)\} |}}_{\phi }$,$\phi$ denotes the vector of maximum likelihood estimates of the parameters $\sigma_{b}^{2} R$, $\sigma_{e}^{2}$ and $\alpha$. Based on the projection matrix, $P = I_{n} - X(X^{T} V^{ - 1} X)^{ - 1} X^{T} V^{ - 1}$, the score statistic using the projection matrix is ${\text{Q}} = {{0.5\{ {\text{y}}}}^{{\text{T}}} {\text{P}}^{{\text{T}}} {\text{V}}^{{ - 1}} {\text{PKP}}^{{\text{T}}} {\text{V}}^{{ - 1}} {{{\text{Py}} \} | }}_{\phi }$, where Q follows $\sum {\lambda_{i} x_{i}^{2} }$, $\lambda$ is the ${\text{n}} \times {\text{n}}$ eigenvalues of matrix ${ 0}{\text{.5V}}^{{ - 1/2}} {\text{PKP}}^{{\text{T}}} {\text{V}}^{{ - 1/2}}$, $x_{i}^{2}$ is the chi-squared distribution with 1 degree of freedom^24^, and p-value could be obtained by Kuonen’s method^25^.

The Genetic Analysis Workshop 18 (GAW18) ^26^ provided the whole genome sequencing data that involved 8,348,674 single nucleotide variations (SNVs), longitudinal phenotype data for hypertension, and related traits in 20 pedigrees. Raw data were processed, and the final sample included 835 individuals. Table 1 displays the descriptive statistics, and Table 2 presents the sample sizes of each family (N = 1389, not 835).

**Table 1:** Descriptive Statistics of phenotype data.

Variable	Exam 1	Exam 2	Exam 3	Exam 6
N	809	578	594	231
Year of exam	1992–1996	1997–2000	1998–2006	2009–2011
Mean age at exam (range)	39.4 (16–94)	42.6 (17–97)	46.5 (18–95)	50.9 (30–81)
Mean SBP (range)	122 (80–216)	125 (90–211)	125 (76–220)	128 (93–233)
Mean DBP (range)	71 (40–123)	72 (43–115)	71 (32–108)	78 (46–126)
Antihypertensive medication (%)	10.05	19.37	28.76	43.67
Hypertension (%)	18.00	29.58	36.58	52.38
Smoking status (%)	22.79	15.92	18.86	11.26

**Table 2:** The sample size of each family by sex.

Pedigree number	Sex		Individual	Pedigree number	Sex		Individual
2	Female	53	107	14	Female	30	60
	Male	54			Male	30	
3	Female	46	98	15	Female	24	57
	Male	52			Male	33	
4	Female	46	97	16	Female	32	59
	Male	51			Male	27	
5	Female	48	91	17	Female	28	57
	Male	43			Male	29	
6	Female	44	88	20	Female	26	51
	Male	44			Male	25	
7	Female	37	89	21	Female	22	50
	Male	52			Male	28	
8	Female	38	84	23	Female	18	46
	Male	46			Male	28	
9	Female	45	81	25	Female	21	44
	Male	36			Male	23	
10	Female	41	83	27	Female	24	44
	Male	42			Male	20	
11	Female	39	76	47	Female	11	27
	Male	37			Male	16	
					**N** = 1389		

Fixed effects are age, sex, smoking, and medications for blood pressure controls (BPMEDS), where SNPs and familial structures are random effects. Dependent variables include systolic (SBP) and diastolic blood pressure (DBP). According to the Shapiro–Wilk normality test, p-values for DBP and SBP are ${2}{\text{.2}} \times {10}^{{ - 16}}$ and ${1}{\text{.35}} \times {10}^{{ - 5}}$, respectively. Hence, Blom's transformation^27^ was applied before the analysis.

The linear mixed effect model for FFBSKAT is

$$ y_{i} = \beta_{{0}} + \beta_{age} age_{i} + \beta_{sex} sex_{i} + \beta_{BPMEDS} BPMEDS_{i} + \beta_{smoke} smoke_{i} + h_{i} + b_{i} , $$

The sample size n is 835. h is a random effect ~ ${\text{N(0}},\tau {\text{K)}}$. ${\text{K}} = {\text{GWWG}}^{{\text{T}}}$, where $G_{n \times m}$ represents every analysis block, and the number of SNVs "m" is 20. $W_{m \times m}$ is the weights for SNVs. b is a random effect ~ ${\text{N(0}}, \, \sigma_{{\text{b}}}^{{2}} {\text{R}}_{{{\text{n}} \times {\text{n}}}} {)}$, where R is kinship correlations. This model was proposed by Svishcheva GR et al. ^12^.

The newly proposed mixed effect model for FGE-SKAT is:

$$ y_{i} = \beta_{{0}} + \beta_{age} age_{i} + \beta_{sex} sex_{i} + \beta_{BPMEDS} BPMEDS_{i} + \beta_{smoke} smoke_{i} + h_{1i} + h_{2i} + b_{i} . $$

G, W, and b are identical to that of the FFBSKAT, where $h_{1}$ and $h_{{2}}$ are random effects, $h_{1}$ ~ ${\text{N(0}},\tau_{{1}} {\text{K}}_{{1}} {)}$ with ${\text{K}}_{{1}} = \rho {\text{GWWG}}^{{\text{T}}}$, $h_{{2}}$ ~ ${\text{N(0}},\tau_{{2}} {\text{K}}_{{2}} {)}$ with ${\text{K}}_{{2}} = {(1 - }\rho {\text{)EGWWG}}^{{\text{T}}} {\text{E}}$, $E_{n \times n}$ is environmental factors. Here denotes smoking status. Let $K = K_{1} + K_{2}$ which is similar to the "rareGE" package by Han Chen^13^. $\uprho =0, 0.1, 0.2, \cdots , 0.9, 1$ (11 values). Since there are 834,030 sliding windows examined, the Bonferroni correction was applied to ensure the most conservative conclusions to avoid the multiple testing issue.

Although the default of the FGE-SKAT software examines only 11 points ($\uprho =$ 0 to 1 by 0.1), results could reveal the patterns of the p-values with respect to the ${\uprho }^{{^{\prime}}}\mathrm{s}$. If running time is not an issue, the user could adopt more points of ${\uprho }^{{^{\prime}}}\mathrm{s}$ in the FGE-SKAT software, such as $\uprho =$ 0 to 1 by 0.01, and the implementation is effortless. In machine learnings and artificial neural networks, the grid search for the optimal hyper-parameters using tenfold cross-validations is a common and powerful technique^28,29^. A well-known regularized regression method, the elastic net^30^, is a convex combination of the ridge^31^ and lasso^32^ regressions. The size of the respective penalty terms is tuned via cross-validations to find the model's best fit. Regardless of the number of scenarios fitted to find the optimal combination, the searching procedure does not adjust for the multiple testing. The elastic net's methodology concept is similar to the FGE-SKAT that combines the FFBSKAT and rareGE via the hyper-parameter $\uprho$. Since the optimal $\uprho$ follows the same strategy, Bonferroni's correction should not depend on the number of grids used to find the optimal $\uprho$. Instead, we adjust for the two joint tests in the FGE-SKAT using two times the Kuonen's method p-value. Finally, the simulation studies demonstrated that the type-I error of the FGE-SKAT is valid under the significance levels 5% and 1%. Therefore, the adjusted minimum p-value was our decision theory.

We conducted a permutation study with one thousand repetitions to obtain the empirical Type-I errors to ensure the validity of the FGE-SKAT. When the phenotypes are randomly permuted without disturbing the genetic components and the family structure, this procedure generated the null distribution. In other words, the phenotypes independent of the set of genetic predictors and other covariates. We arbitrarily selected the first 50 SNVs on chromosomes 1, 3, and 5 for permutations from the GAW18 data. As a result, there are four sliding windows for each chromosome. Permutation studies evaluated both 5% and 1% nominal levels.

Regarding the power study, we choose the first 50 SNVs on chromosome 5 for simulations and randomly picked the 16th SNV to generate the SBP. The name of the SNV is X5_13329, and we assumed the recessive disease model to simulate the trait. Hence, the SBP would be elevated if the genotype of X5_13329 is 2, and the SBP would be normal if the genotype is 0 or 1. The environmental effect is the smoking status (yes vs. no). In this way, the first and the second sliding windows contain the genetic effect. However, the third and fourth sliding windows do not cover the main genetic effect but have linkage disequilibrium (LD). We examined four scenarios for each disease model in the FFBSKAT and FGE-SKAT with one thousand repetitions. The first scenario is the pure genetic effect without the smoking effect on the trait, where we expect that the FFBSKAT and FGE-SKAT should demonstrate similar statistical power. In the second scenario, we want to ensure that the FGE-SKAT would not detect the wrong environmental effect when the genetic effect is absent. Thus, the SBP only depends on the smoking variable but not the SNV. In the third scenario, we want to show that the FGESKAT could discover gene-environment interactions, but the FFBSKAT failed in this situation. Therefore, only the SNV by smoking interaction contributes to the SBP variations. Finally, we simulated a weaker interaction effect with some environmental and genetic effects to show dose–response in power evaluations such that we could have more confidence in the performance of the new strategy.

## Results

We summarize the permutation results in Table 3, and the first column is the genetic disease model used in the FGE-SKAT software. Among the three chromosome results noted in the second column, the two methods demonstrate valid Type-I errors for all sliding windows well under the nominal level threshold of 0.05 or 0.01. Relative comparisons were not consistent since the FGE-SKAT may be randomly higher or lower than that of the FFBSKAT.

**Table 3:** Permutation studies for Type-I errors.

Alpha	Chr	Window 1		Window 2		Window 3		Window 4	
		FFBSKAT	FGE-SKAT	FFBSKAT	FGE-SKAT	FFBSKAT	FGE-SKAT	FFBSKAT	FGE-SKAT
0.05	1	0.056	0.045	0.056	0.054	0.044	0.054	0.048	0.052
0.05	3	0.045	0.048	0.054	0.049	0.046	0.054	0.044	0.0510
0.05	5	0.032	0.044	0.034	0.045	0.04	0.053	0.041	0.044
0.01	1	0.012	0.01	0.009	0.01	0.006	0.009	0.003	0.006
0.01	3	0.007	0.009	0.009	0.01	0.011	0.014	0.011	0.013
0.01	5	0.009	0.01	0.006	0.009	0.01	0.012	0.007	0.011

The first column, "Alpha" represents the nominal significance level and the second column, "Chr." represents the chromosome number.

Table 4 shows the results of the power study. The first column is the genetic disease model used in the FGE-SKAT software. The second column indicates the four mean SBPs among non-smokers without the SNV, non-smokers with the SNV, smokers without the SNV, and smokers with the SNV. The standard deviation is 10 for the four groups. The first scenario (120,180,120,180) means that the recessive disease model of SNV (X5_13329 = 2) contributes to the elevated SBP, but the SBP is not affected by the smoking status. Regardless of the disease model used in the FGE-SKAT software, the FFBSKAT and the FGE-SKAT showed similar statistical power.

**Table 4:** Simulations for statistical power.

Model	Scenario	Window 1		Window 2		Window 3		Window 4	
		FFBSKAT	FGE-SKAT	FFBSKAT	FGE-SKAT	FFBSKAT	FGE-SKAT	FFBSKAT	FGE-SKAT
Dom	120,180,120,180	0.14	0.03	0.624	0.295	0.999	0.992	0.985	0.92
Dom	120,120,180,180	0.049	0.053	0.05	0.046	0.054	0.056	0.05	0.051
Dom	120,120,120,180	0.027	0.367	0.025	0.462	0.013	0.172	0.008	0.06
Dom	120,120,150,180	0.057	0.187	0.061	0.189	0.033	0.078	0.025	0.046
Add	120,180,120,180	0.151	0.036	0.617	0.287	0.996	0.992	0.98	0.913
Add	120,120,180,180	0.054	0.048	0.05	0.047	0.04	0.034	0.043	0.042
Add	120,120,120,180	0.018	0.341	0.02	0.461	0.006	0.178	0.006	0.049
Add	120,120,150,180	0.041	0.191	0.035	0.188	0.023	0.066	0.021	0.045
Rec	120,180,120,180	1	1	1	1	0.019	1	0.019	1
Rec	120,120,180,180	0.056	0.048	0.05	0.05	0.049	0.047	0.049	0.045
Rec	120,120,120,180	0.99	1	1	1	0.005	1	0.005	1
Rec	120,120,150,180	0.746	1	1	1	0.022	1	0.022	1
Rec	120,140,120,140	0.999	0.999	1	1	0.059	0.989	0.059	0.988
Rec	120,120,140,140	0.036	0.038	0.033	0.037	0.037	0.041	0.037	0.04
Rec	120,120,120,140	0.458	0.964	0.999	1	0.028	0.961	0.028	0.959
Rec	120,120,130,140	0.16	0.0457	0.721	0.999	0.04	0.0471	0.04	0.0469

The four numbers listed in the scenarios column are the four means of the normally distributed SBP with a standard deviation of 10 for four groups (non-smokers without the SNV, non-smokers with the SNV, smokers without the SNV, and smokers with the SNV).

The second scenario (120,120,180,180) means that the SBP is higher, about 180 only when the smoking effect is present, but this value is not affected by the SNV. Both methods have valid results since the chance of detecting such erroneous information is less than 5%.

The third scenario (120,120,120,180) means that the mean SBP could be 180, higher than the other three groups only when the SNV by smoking interaction effect is present. The FGE-SKAT demonstrated much superior power to the FFBSKAT. This phenomenon explains the need for our new approach in genetic research.

The fourth scenario (120,120,150,180) has a weaker interaction effect and an extra genetic effect than the third scenario. Therefore, the FFBSKAT has small power but inferior to the FGE-SKAT. Simultaneously, we observed the dose–response effect since the FGE-SKAT has smaller power than itself in the third scenario.

When the disease model of the FGE-SKAT is correctly specified in the analysis, the power is almost 100%, and the relative comparisons are not clear. Therefore, we added additional four scenarios at the bottom of Table 5 when the elevation of SBP is 140 but not 180. The results revealed similar patterns, which further confirms the superior performance of the FGE-SKAT even when the genetic or interaction effect is much weaker.

**Table 5:** The most significant genes identified by both methods for normalized DBP.

Chromosome	FBSKAT		FGE-SKAT	
	Gene_Seq	UniGene	Gene_Seq	UniGene
Chr3	LOC105374165	0	LOC105374165	0
Chr5	–	–	0	0
Chr7	CACNA2D1	CACNA2D1	CACNA2D1	CACNA2D1
Chr9	–	–	0	0
Chr13	–	–	0	0

**Figure 1:**
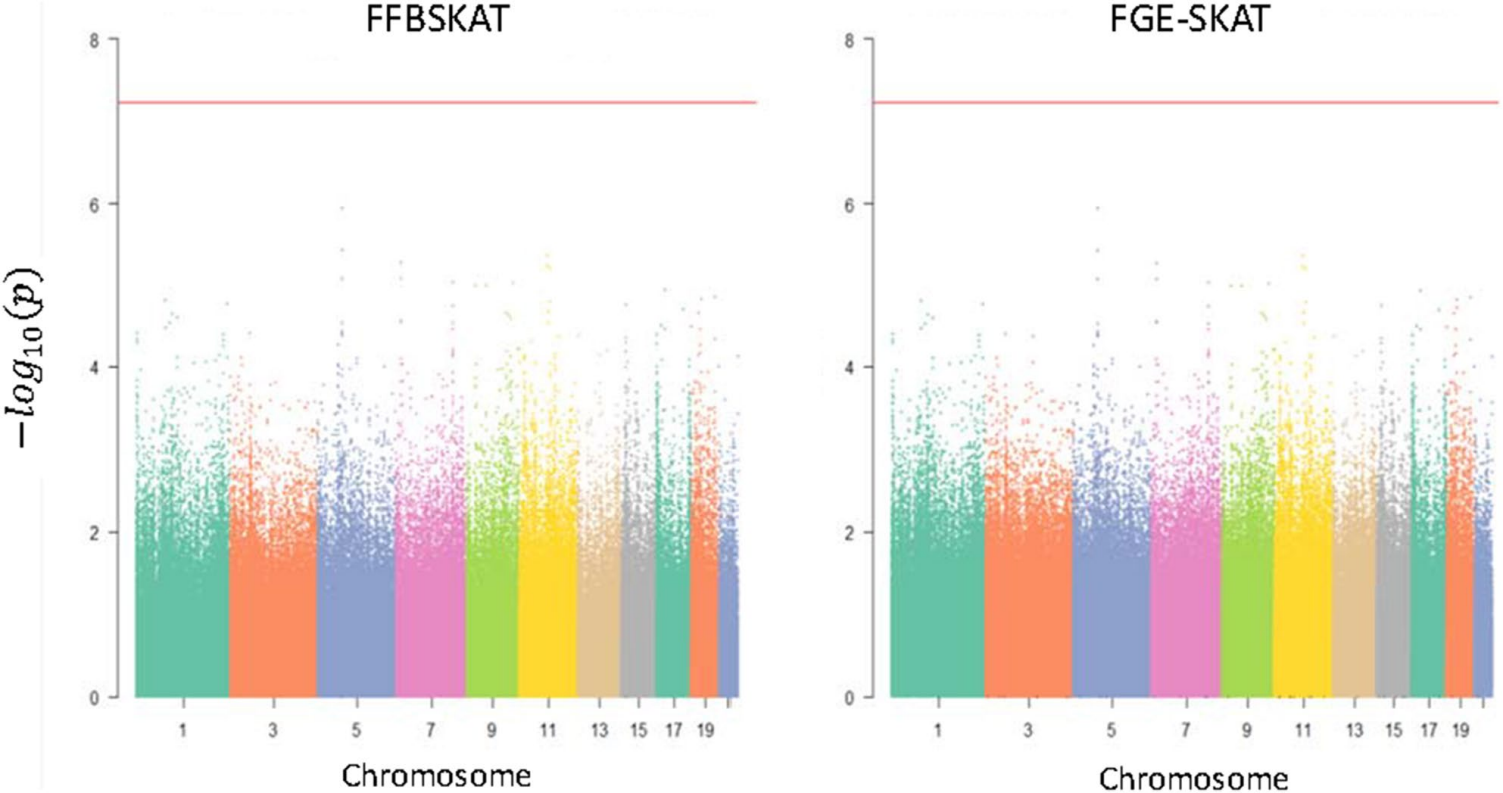
Manhattan plot for normalized DBP.

After the validity and performance of the FGE-SKAT are confirmed, this approach is applied to the GAW18 data. Results of GWAS are displayed in Fig. 1. The Manhattan plots of normalized DBP revealed that all p-values in a scale of –log10 are lower than the red line, indicating that all p-values are over ${5}{\text{.995}} \times {10}^{{ - 8}}$, which is adjusted for the Bonferroni's correction with 834,030 sliding windows using $\mathrm{\alpha }=0.05$($\frac{0.05}{834030}=5.995\times {10}^{-8}$). Therefore, the smallest p-values in Fig. 1 were selected to be compared with the analyses using the original DBP, but the results were similar and shown in Online Appendix A.

In Table 5, consistent results were found on chromosomes 3, 5, 7, 9, and 11. In the upper part of Table 6, we identified two segments on chromosome 7 from 18,473,528 to 18,478,318 base pairs and 18,475,056 to 18,479,387 base pairs.

**Table 6:** Top 10 smallest p-values for normalized DBP and SBP.

CHR	Trait	Genomic region	FFBSKAT P-value	FGE-SKAT P-value
5	DBP	50,274,970–50,279,007	3.79246E−06	3.79246E−06
5	DBP	50,318,550–50,318,624	1.15102E−06	1.15102E−06
5	DBP	50,319,835–50,323,204	8.42894E−06	8.42894E−06
7	DBP	18,473,528–18,478,318	8.49578E−06	8.49578E−06
7	DBP	18,475,056–18,479,387	5.29517E−06	5.29517E−06
7	DBP	132,160,189–132,163,619	9.36002E−06	9.36002E−06
9	DBP	133,316,470–133,319,343	9.65182E−06	9.65182E−06
11	DBP	77,592,371–77,595,131	5.82584E−06	5.82584E−06
11	DBP	77,593,756–77,596,971	4.43435E−06	4.43435E−06
11	DBP	82,432,685–82,435,840	6.19042E−06	6.19042E−06
7	SBP	139,953,680–139,955,405	1.82164E−07	1.66733E−07
7	SBP	139,954,850–139,956,291	9.09435E−08	9.09435E−08
7	SBP	139,959,269–139,961,904	1.83966E−07	1.82164E−07
7	SBP	142,258,881–142,262,340	7.67391E−07	7.67391E−07
7	SBP	143,609,266–143,611,641	6.65998E−07	6.65998E−07
7	SBP	145,963,864–145,967,584	1.87147E−07	1.83966E−07
7	SBP	146,913,363–146,918,656	5.85388E−07	5.85388E−07
7	SBP	146,916,497–146,920,752	2.1425E−07	2.1425E−07
7	SBP	146,918,731–146,922,053	2.5671E−07	2.5671E−07
7	SBP	148,901,779–148,904,624	4.85975E−07	4.85975E−07

**Figure 2:**
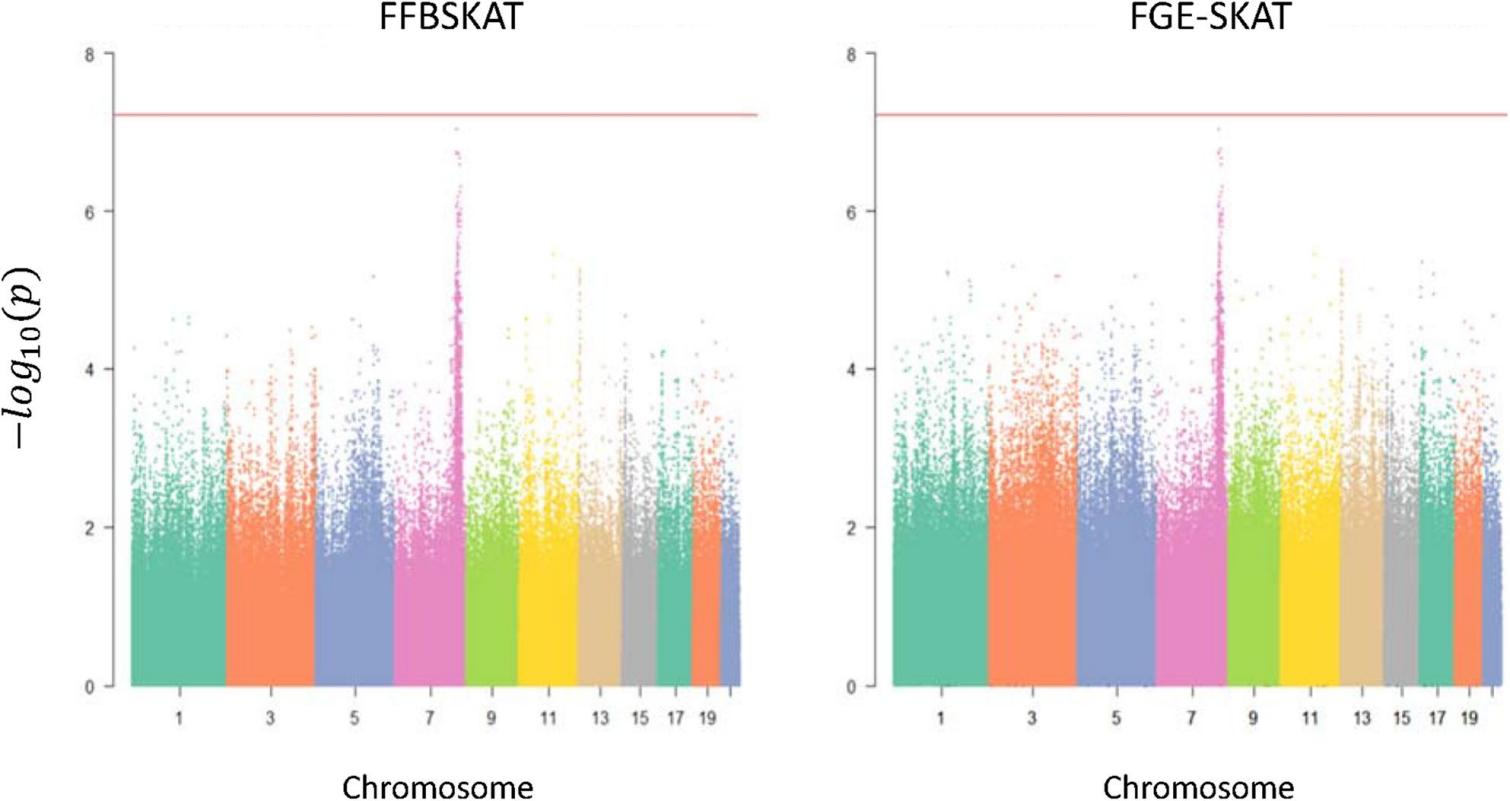
Manhattan plot for normalized SBP.

In Fig. 2, results of the normalized SBP also suggest non-significant p-values since all p-values in a scale of – log10 are lower than the red line. The FGE-SKAT yielded more signals in many genetic regions than that of the FFBSKAT since more points are over 4 in the right panel. Among the smallest p-values, 10 of the segments are further examined, where both FFBSKAT and FGE-SKAT identified the same regions. Chromosome 7 has a peak with the smallest p-value in the lower part of Table 4. Even though the p-values do not exceed Bonferroni's threshold, the associations are indicative. Note that the results of the original SBP are similar and shown in Online Appendix B.

## Discussions

The FGE-SKAT is the first proposed in the family-based SKAT method to detect genetic environment interactions based on rare variations. This research also provides a free R function that facilitates the implementation. The manual clearly describes the usage of FGE-SKAT with similar settings in the FFBSKAT. An automatic pipeline using the R code with an illustrative example facilitates the implementation of this new approach.

In the application of GAW18 data, FGE-SKAT identified the most significant genetic region with interaction signals. Note that all analyses were based on Blom's transformation of SBP or DBP. However, analyses were also conducted for the original BP values. Although the p-values were much more significant than the normalized results, the regions discovered were very similar and hence not shown. Besides, using normalized outcomes avoids concerns regarding the validity of analyses.

The FGE-SKAT results were based on the smallest p-value among 11 points of $\mathrm{\rho {^{\prime}}}\mathrm{s}$. In many regions, the p-values of FFBSKAT and FGE-SKAT are identical. This phenomenon suggests that interactions are absent with $\uprho =1$. It is worth noting that the most significant results are mostly seen when $\uprho =0\mathrm{ or }1$. Results of $\uprho =0$ are proof that interactions alone could enhance the chance of discovering significant regions.

In this research, the sliding window is chosen as 20 SNVs, with 10 SNVs shifted for the next window. If more biological information is available, the parameters could be modified to increase statistical power^12^. The user could easily change the settings of sliding windows in the FGE-SKAT software. Besides, this research uses SKAT settings with minor allele frequency follows Beta distribution (1,25). This method's kernel function is linear, but researchers could also adopt polynomial or Identity of State (IBS) for the kernel functions in the FGE-SKAT software.

Since mixed models are used in family-based studies as well as in studies with unrelated samples (Kinship replaced by GRM), the FGE-SKAT has the potential to be extended using other strategies such as efficiently controlling for case–control imbalance and sample relatedness in large-scale genetic association studies^33^ (SAIGE: https://github.com/weizhouUMICH/SAIGE).

This work is based on samples from the Genetic Analysis Workshop 18 (GAW18). The samples were longitudinal, and the majority of participants had three measurements collected at approximately 5-year intervals. Datasets included systolic and diastolic blood pressure measurements from a human whole-genome sequencing (WGS) study. Thus, this research is dealing with human data.

Evaluations of robustness for this approach against gene-environment correlation and miss-specified environmental main effects require a tremendous effort. It is cumbersome research when several advanced methods are compared under more complicated scenarios. On the other hand, this issue is an excellent topic for future research to examine further such impact for FFBSKAT, rareGE, FGE-SKAT, and other existing methods.

The FGE-SKAT deals with one environmental factor. If there are multiple factors, one could apply the FGE-SKAT repeatedly and control for multiple testing. Extending this new strategy to accommodate multiple factors further is also a promising future research plan.

Although the deep learning model^34^ has demonstrated extraordinary prediction abilities, this approach requires tons of training samples with available features and outcomes. Besides, the computational burden is high. If the quality of training samples is not guaranteed, the model performance may not be satisfactory. In contrast, our statistical approach does not require these assumptions and is ready to use with a satisfying speed.

## Supplementary Information

Supplementary Information.
