# Supplementary material for: Family-based gene-environment interaction using sequence kernel association test (FGE-SKAT) for complex quantitative traits
Source: Sci Rep. 2021 Apr 1;11:7431. doi: 10.1038/s41598-021-86871-2 (PMC8016937; doi:10.1038/s41598-021-86871-2)

Supplementary Materials

Restricted Maximum Likelihood (REML) for FGE-SKAT

APPENDIX A:

Figure A1: Evaluations of Type- I Error with 10000 repetitions


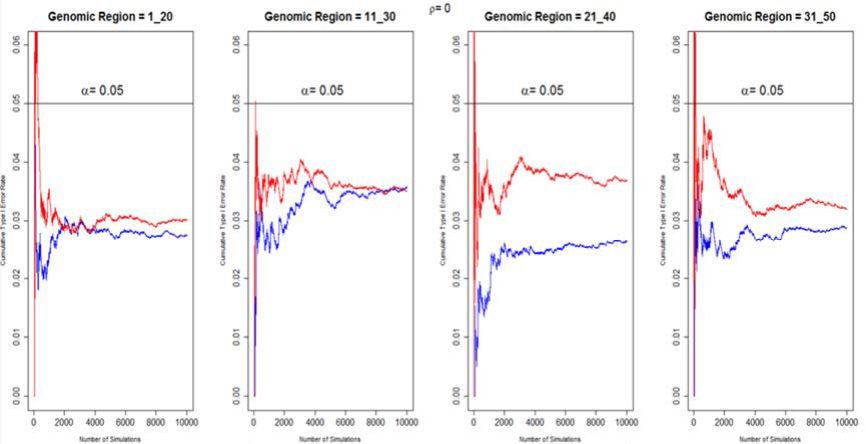


Figure A2: Evaluations of Type- I Error with 10000 repetitions


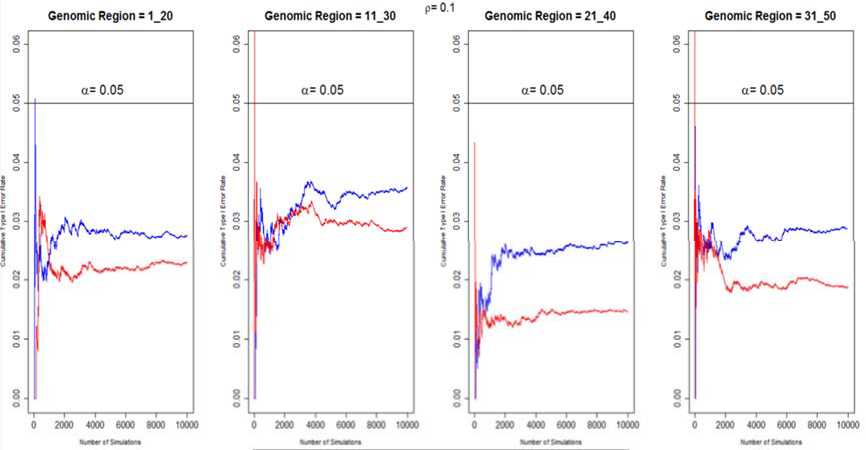


Figure A3: Evaluations of Type- I Error with 10000 repetitions


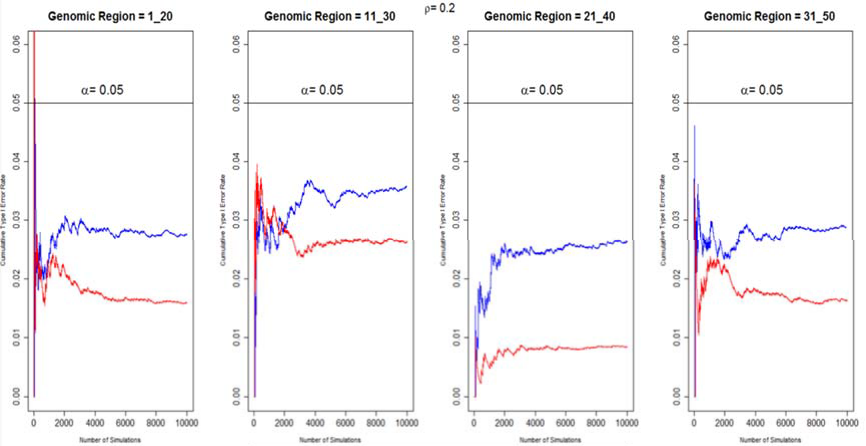


Figure A4: Evaluations of Type- I Error with 10000 repetitions


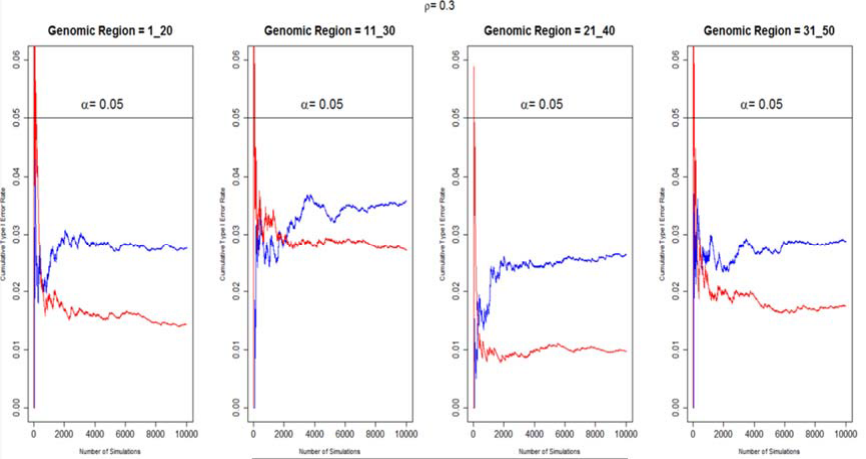


APPENDIX A:

Figure B1: Manhattan plot for original DBP


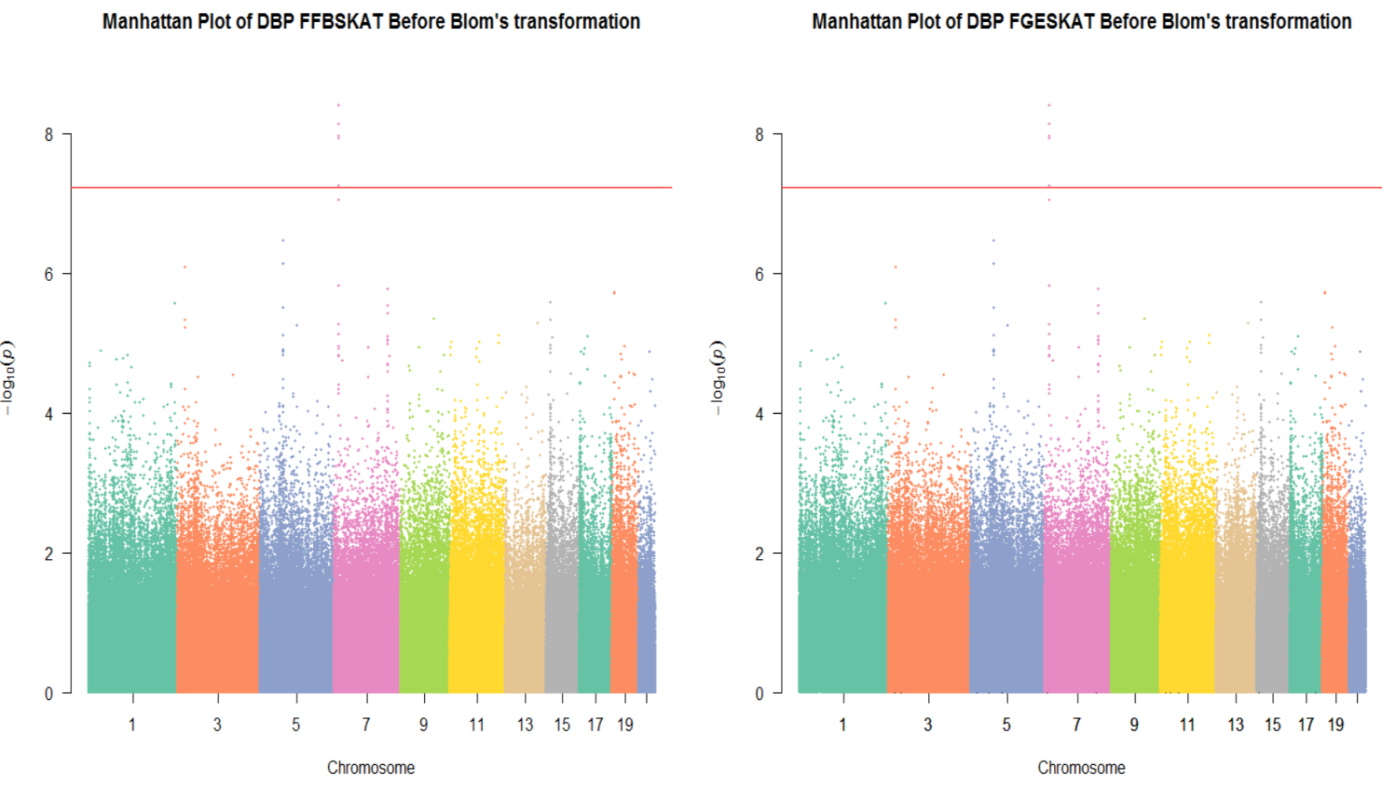


APPENDIX B:

Figure C1: Manhattan plot for original SBP


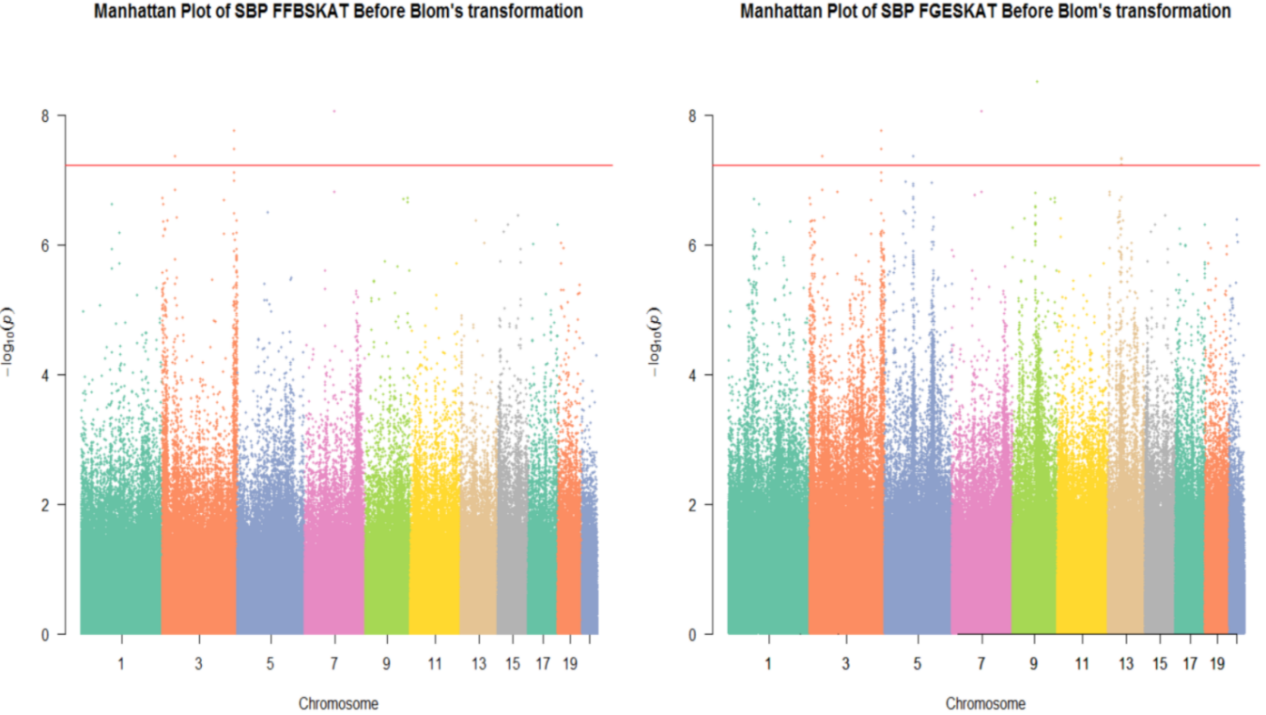

Supplement: Supplementary file 1 — Supplementary Information. [file 41598_2021_86871_MOESM1_ESM.docx]
